# IMU-Based Real-Time Estimation of Gait Phase Using Multi-Resolution Neural Networks

**DOI:** 10.3390/s24082390

**Published:** 2024-04-09

**Authors:** Lyndon Tang, Mohammad Shushtari, Arash Arami

**Affiliations:** 1 Department of Mechanical and Mechatronics Engineering, University of Waterloo, Waterloo, ON N2L 3G1, Canada; smshushtari@uwaterloo.ca (M.S.); arash.arami@uwaterloo.ca (A.A.); 2 KITE Institute, University Health Network, Toronto, ON M5G 2A2, Canada

**Keywords:** gait phase estimation, gait variability, inertial measurement unit

## Abstract

This work presents a real-time gait phase estimator using thigh- and shank-mounted inertial measurement units (IMUs). A multi-rate convolutional neural network (CNN) was trained to estimate gait phase for a dataset of 16 participants walking on an instrumented treadmill with speeds varying between 0.1 to 1.9 m/s, and conditions such as asymmetric walking, stop–start, and sudden speed changes. One-subject-out cross-validation was used to assess the robustness of the estimator to the gait patterns of new individuals. The proposed model had a spatial root mean square error of 5.00±1.65%, and a temporal mean absolute error of 2.78±0.97% evaluated at the heel strike. A second cross-validation was performed to show that leaving out any of the walking conditions from the training dataset did not result in significant performance degradation. A 2-sample Kolmogorov–Smirnov test showed that there was no significant increase in spatial or temporal error when testing on the abnormal walking conditions left out of the training set. The results of the two cross-validations demonstrate that the proposed model generalizes well across new participants, various walking speeds, and gait patterns, showcasing its potential for use in investigating patient populations with pathological gaits and facilitating robot-assisted walking.

## 1. Introduction

Lower-limb exoskeletons serve as valuable tools to enable motor-impaired individuals to walk again, for instance, those who have had a spinal cord injury or stroke [1]. In rehabilitation and assistive applications for incomplete spinal cord injuries (iSCI), the wearer may have partial control over their muscles, but lack the muscular strength to hold an upright posture, support their full weight on their stance leg during walking, or effectively control their swing leg to maintain a stable gait [2].

A major challenge that is preventing the adoption of exoskeletons in iSCI rehabilitation is synchronizing the exoskeleton with the user. Controllers that impose a predefined motion on the user, independent of their actions, have been shown to reduce active user engagement during rehabilitation, which can undermine the progression towards independent walking [3]. In the assist-as-needed paradigm, the controller detects the intentions of the user and supplies assistive forces only when necessary, using controllers such as adaptive feedforward control [4,5,6,7,8,9] or impedance tracking control [10,11,12,13,14,15]. The intentions of the user may be decoded from the kinematics of the limbs [6,15,16], or measured through muscle activations [7,8,9] and interaction torques between the user and the exoskeleton [17]. In many assist-as-needed controllers, gait phase, a quantity that defines how far along a person is in their stride, is used as a key control variable for synchronizing the controller with the user [10,18,19,20].

Gait phase is a useful variable for the analysis and modelling of human walking, as well as for the design of cooperative control systems for assistive walking technologies [5,18,19,20]. In many controllers, it serves as a proxy variable for time that depends entirely on the state of the user’s body, and can be sped up or slowed depending on the user’s gait pattern [21,22]. Therefore, real-time gait phase estimators have emerged as a necessary technology for the application of these controllers.

Gait phase estimation is a challenging problem due to the differences in gait patterns across individuals and walking speeds, as well as the ambiguous definition of gait phase. A continuous variable describing the subject’s position within the gait cycle can be unambiguously assigned when the gait pattern remains consistent throughout each stride. Conversely, variations in timing and gait kinematics lead to uncertainty about how much of the stride has been executed and how much remains.

Previous studies have defined gait phase using various kinematic variables so that it monotonically increases throughout the progression of a stride, and well-defined events in the gait cycle, such as the heel strike, correspond to the same gait phase value. The phase of the hip flexion angle and its derivative in state-space phase portrait [10,23] or its integral [24] satisfies the properties of gait phase outlined above [25]. Other studies have used the cyclic patterns in the knee and hip flexion angles to create a mapping to a gait phase variable that increases linearly from 0 to 1 between heel strikes [19,26,27,28,29,30]. Some studies that use this gait phase mapping use piecewise linear mappings to separate the swing and stance phase of the gait cycle [31,32].

The sensors used to estimate gait phase vary depending on the application. In studies where the estimator inputs are hip and knee flexion angles, the joint angles are measured by encoders [19,24,26,28,30], or two inertial measurement units (IMUs) fixed on the proximal and distal segments forming the joint [27,33]. Certain studies have used a single IMU placed on the subject’s shank [31,34] or thigh [35] to estimate the gait phase. Contact sensors such as heel pressure sensors [18,19,27] or capacitive sleeves that measure changes in leg muscle shape have also been used [36]. In some cases, these sensors may be uncomfortable to wear because they cross the joint, and restrict motion to an axis of rotation that may not be aligned with the true axis of rotation if fitted poorly. In comparison, IMU sensors placed on either side of a joint can be used to measure joint angles without interfering with the range of motion [27]. This paper proposes a gait phase estimator that uses only IMUs fixed on the thigh and shank of each leg, removing the need for obtrusive sensors. The resulting challenge of using IMUs is that the joint angles, which have been used as regressors for gait phase in many other estimators [19,24,26,28], are no longer directly measured but are estimated from angular velocities and linear acceleration measured in the moving frame of IMUs.

The algorithms used to estimate gait phase are mostly time-varying regression models that adapt to changes in the gait pattern. Adaptive frequency oscillators (AFOs) [36,37,38] and extended Kalman filters (EKFs) [19,39] have been used to form a mathematical model of the patterns in the inputs from which the phase can be derived. These methods are suitable when the walking patterns are consistent and repetitive; however, their performance when the user deviates from the established pattern is limited by their simplicity.

Other studies have solved the gait phase estimation problem directly by using artificial neural networks (ANNs) to learn the mapping from sensor measurements directly. These ANN models capture the time-varying patterns of walking by feeding a window of time-delayed inputs into a feedforward network [31] or convolutional neural network (CNN) [28], or by using a recurrent neural network (RNN) [33] or a derivative such as the long short-term memory (LSTM) [27,32,33,34] or time-delay neural network (TDNN) [26]. Gait phase estimation is a many-to-one regression problem, with a weak definition that relates sensor data to gait phase. Deep neural networks, including CNNs, are capable of capturing and storing the complex mappings between raw sensor data and gait phase, even in the presence of of uncertain labels and edge cases, unlike the previously mentioned AFO and EKF algorithms.

CNNs in particular, produce translationally invariant features, which, in the context of time-series regression, means that the features are time-invariant. This leads to a robust gait-phase estimator that handles temporal variations in sensor data due to different walking speeds, and variations in key gait event timings within the gait cycle.

Three studies that used only IMUs to estimate continuous gait phase, [31,33,35], relied on data-driven algorithms. The accuracy and generalizability of these data-driven regression models depend on the diversity of the gait patterns in the dataset. The dataset used in [33] to train an RNN had seven participants in the dataset, walking with a range of walking speeds and stride lengths. Weigand et al. used a dataset of 12 participants walking at their preferred pace, then transitioning to climbing a staircase [31]. Zhang et al. also used seven participants in the dataset, with six different walking speeds ranging from 0.50 to 1.75 m/s [35]. Despite capturing some variations of gait speed/stride length in those studies, none provide a robust estimation of gait phase across different gait conditions such as intermittent changes in speed, and semi-pathological gait, for instance, with asymmetric strides. We trained and evaluated our estimator on a dataset that includes such conditions to demonstrate the feasibility of robustly estimating gait phase using IMU sensors under varying walking conditions.

To achieve robustness across a range of walking conditions and gait patterns, other studies have used time windows of input features [26,27], allowing the estimator to consider a time history of regressors that carries gait patterns that are unique to the individual, and the walking conditions. This work extends the idea of time windowing by using multi-rate processing to efficiently capture long time windows without compromising the accuracy or computational complexity of the estimator. We designed a custom CNN architecture to estimate gait phase using windows of data sampled at different rates. In this study, the justification of using multiple sampling rates in a CNN-based gait phase estimator are evaluated by comparing the performance of an estimator with and without this modification.

This study has two main contributions: (1) Development of a novel, multi-rate CNN estimator for robust estimation of gait phase from IMU data; (2) The proposed estimator was trained and evaluated on various walking conditions, including different stride lengths, walking speeds, intermittent stops, and asymmetric gaits.

## 2. Materials and Methods

The following section describes the data collection procedure, processing of the IMU data, and the proposed gait phase estimator algorithm.

### 2.1. Experiment Setup

**Figure 1:**
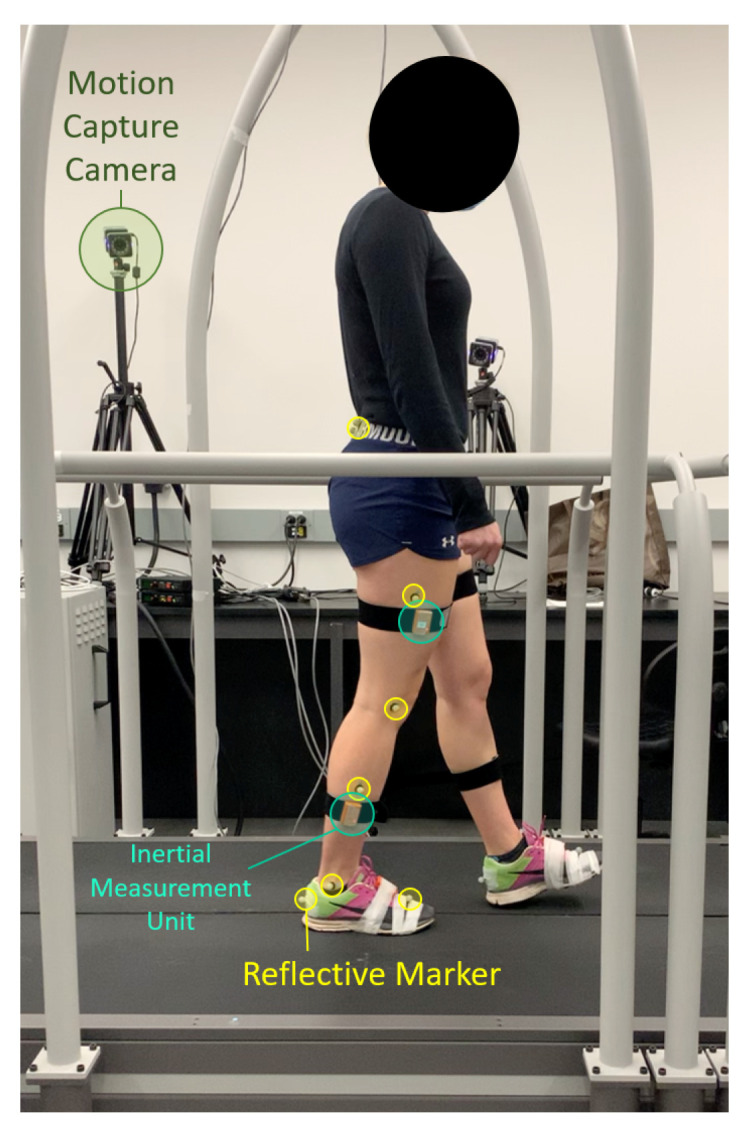
Kinematics are recorded with the motion capture cameras (green) and retro-reflective markers (yellow). Thigh and shank acceleration and angular velocity are measured with IMUs placed highlighted (teal).

Participants walked on an instrumented split belt treadmill (Bertec, Columbus, OH, USA). Kinematics were measured using a motion capture comprising 8 Vero cameras (Vicon, Oxford, UK) and 16 retro-reflective markers (see Figure 1) placed according to the Plug-in gait convention (Vicon, UK) at a rate of 100 Hz. Four IMUs (Xsens, Enschede, The Netherlands) were placed on the lateral aspect of the participant’s mid-thighs and calves (see Figure 1). Each IMU provides real-time measurements of acceleration, angular velocity, and orientation of the sensor frame at a sampling rate of 100 Hz.

### 2.2. Experiment Protocol

Sixteen participants (age: 28 ± 4 years, body mass: 72.6 ± 18.5 kg, height: 175 ± 8.0 cm, 8 females and 8 males) with no known musculoskeletal disease or sensory and motor impairments were recruited. Each participant provided informed consent prior to the experiment. The study protocol and procedures were approved by the University of Waterloo Clinical Research Ethics Committee (ORE#41794) and conformed with the Declaration of Helsinki.

Participants walked on the treadmill for 13 min under 8 different conditions. In the first 4 conditions, each participant walked at their preferred (normal) stride length, followed by short strides, long strides, then back to normal strides, with the treadmill speed set at 0.8 m/s for 45 s for each condition. In the fifth condition, the treadmill speed increased from 0.1 to 1.9 m/s at a constant rate then decreased back to 0.1 m/s. The sixth condition induced sudden changes in walking speed by holding the speed at 0.2, 0.4, and 0.8 m/s and stepping up the speed. The seventh condition introduced asymmetrical gait patterns by slowing down the left treadmill belt, then the right belt, from 0.8 to 0.4 m/s. The last condition alternated between a full stop and walking at 0.8 m/s. More details about the treadmill protocol can be found in [26]. Prior to the experiment, participants were asked to perform hip and knee flexion and extension movements in the sagittal plane to obtain plane-restricted motions for functional calibration of the IMUs.

### 2.3. Data Labeling and Preprocessing

The target gait phase labels for each leg, $\phi_{R}$ and $\phi_{L}$ for the right and left legs, respectively, were assigned so that they linearly increase from 0 to 1 between two consecutive heel strikes, as in [19,26,27,28,29]. The linear gait phase representation suffers from a discontinuity at the transition boundary between 0 and 1, so it is ill-suited for supervised learning with gradient descent algorithms. To avoid the discontinuity, the linear representation was converted to sinusoidal vector representations, $y_{1R}$ and $y_{2R}$ for the right leg, and similarly for the left [19,26,27,28,29].

(1) $$\left[\begin{array}{c} y_{1R}\\ y_{2R}\\ y_{1L}\\ y_{2L} \end{array}\right]=\left[\begin{array}{c} cos(2\pi \phi_{R})\\ sin(2\pi \phi_{R})\\ cos(2\pi \phi_{L})\\ sin(2\pi \phi_{L}) \end{array}\right].$$

Heel strikes were labelled using the heel marker motion capture data. Heel strikes were identified at time instances where the vertical distance of the heel marker from the ground was a local minimum and there was a peak in the vertical acceleration of the heel. A linearly increasing function was assigned between heel strikes, except when consecutive instances were separated by more than 3 s, indicating that the participant was standing and, therefore, the gait phase was not clearly defined.

Functional calibration was applied in post-processing to align each IMU’s frame (sensor frame) with a body-fixed coordinate frame (segment frame) that is consistent across participants, resulting in sensor measurements that are robust to different sensor placement. The functional calibration procedure used movements such as knee flexion–extension, and methods derived from [40], to determine the transformation between each sensor frame and its corresponding anatomical frames. In a real-time application of the proposed estimator, this procedure should be performed every time the user puts on the IMUs.

### 2.4. Gait Phase Estimator Model

**Figure 2:**
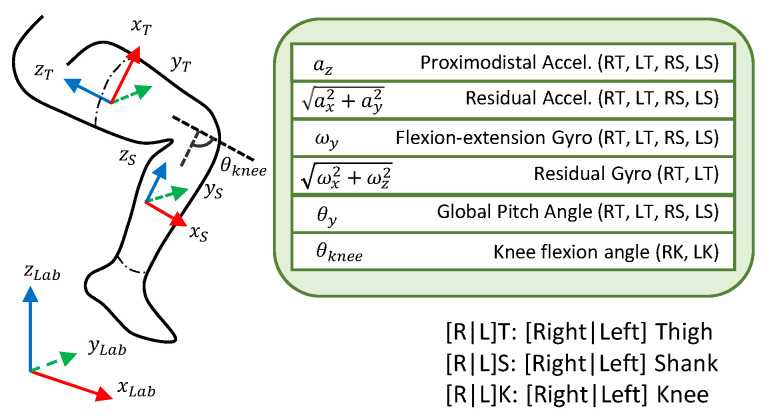
The input features are a combination of acceleration, angular velocities, and pitch angles that correspond to motion in the sagittal plane, measured by the IMUs. There are additional features for the residual components of acceleration and angular velocity, as well as the knee flexion angle.

The gait phase estimator takes features that are derived from the IMU data as inputs, and outputs a sinusoidal representation of the gait phase for each leg. This data-driven estimator minimizes the mean squared error (MSE) loss function between the target and predicted gait phase. The inputs we used were a combination of the knee flexion angles and the thigh and shank accelerations, angular velocities, and orientations, which are detailed in Figure 2. In a real-time implementation of the proposed estimator, all of the input features can be computed online from functionally calibrated IMUs.

The thigh orientation was approximated by the pitch angle of the thigh frame. This feature plays a similar role to the hip flexion angle used in other studies when the torso is in an upright position. The knee flexion angle was estimated by computing the pitch angle of the relative orientation of the shank to the thigh from the computed rotation matrices of each segment obtained from IMUs that have been functionally calibrated.

A fourth-order Butterworth low-pass filter with a cut-off frequency of 10 Hz was applied to the accelerometer data to reduce noise. The acceleration in the segment-aligned z-axis captures normal acceleration due to the movement of the thigh and shank during the swing phase and gravitational acceleration, as well as the acceleration due to the heel strike. The onset of these events corresponds to significant and distinct periods in the gait cycle, so the channel is an effective feature for gait phase estimation. Acceleration in the segment-aligned xy-plane was found to be highly variable between strides and across individuals. Rather than omitting the residual components of acceleration, the magnitude of the acceleration in the xy-plane was used as an input since the specific direction in the xy-plane is not important.

Similarly, the angular velocity was decomposed into two components: the angular velocity about the axis of flexion, and the residual component that is perpendicular to the axis of flexion. The angular velocity about the flexion axis and the magnitude of the orthogonal residual component were used as two additional inputs to the model. It is worth mentioning that the residual component of the angular velocity of the shank, which is orthogonal to the knee flexion angular velocity, was found to be noisy. Therefore, only the residual angular velocity from the thigh IMUs were used (see Figure 2).

A feedforward deep neural network was designed to estimate gait phase, motivated by recent studies that used artificial neural networks to create a robust mapping from the complex and highly variable kinematics of walking to gait phase [26,27,28,33,34], ground reaction dynamics, and joint torques [41,42].

Time dependencies were captured in the model by feeding a sliding window of time-delayed features as inputs, allowing the model to adapt to different gait patterns while disregarding strides from the distant past when estimating the current gait phase. The sliding window inputs increase the robustness and generalizability of the model and increase the input dimensionality, which decreases the likelihood of an ambiguous single-input to multiple-output mapping between the input features and the gait phase.

**Figure 3:**
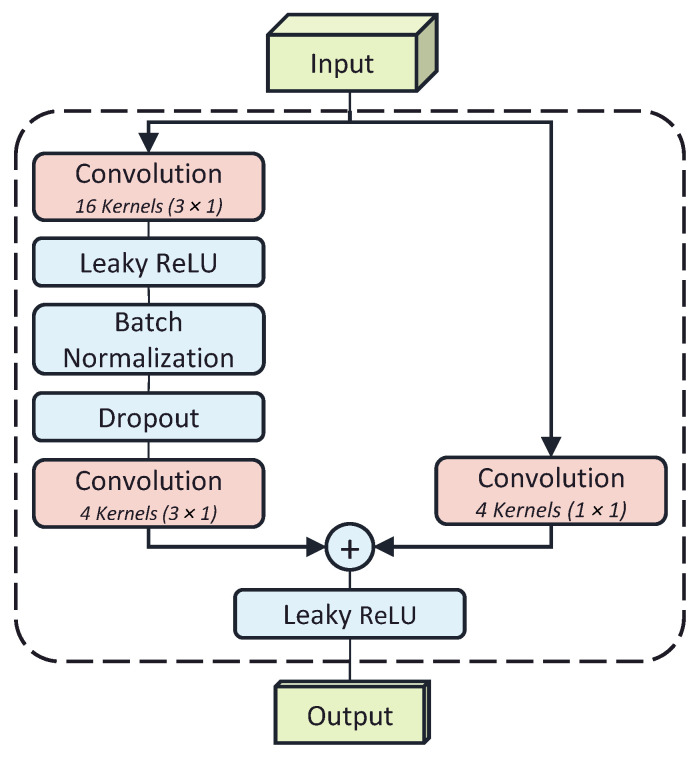
Each residual block contains 2 convolution layers with a 3 × 1 kernel, followed by a batch normalization layer. The first convolution has a leaky ReLU activation and a dropout layer. The residual connection passes through a 1 × 1 convolution layer to reshape the inputs to match the tensor sizes at the addition layer.

The model uses residual convolutional layers to generate feature maps from the input window (see Figure 3). Each layer has 16 convolving kernels of size 3 × 1 along the time axis. The convolutional layers are followed by batch normalization with a mean and variance decay of 0.1, Leaky ReLU activation with a scale of 0.01, and a dropout layer with a rate of 0.5. To reduce the model size and complexity, each block feeds into a 1 × 1 convolution layer with 4 kernels. The dilation factor of each layer of residual blocks exponentially increases with a base rate of 2.

The 1D kernels in the residual layers preserve the time-channel meanings of the feature map in the deep layers. The original height and width of the feature map are preserved by padding the feature map during the convolution. In the final layers, the channel dimension is collapsed via a 16 kernel 1 × 20 convolutional layer with no padding to obtain a single-channel time signal. The resulting output is flattened and fed into a fully connected regression output layer.

Our proposed convolutional neural network is a multi-rate architecture that receives input windows of features sampled at two different rates, capturing compact representations of long time histories of the input features by downsampling. To compensate for the low bandwidth due to downsampling, the most recent data from the sensors is sampled at a higher rate.

In this study, the problem of compactly processing long-duration time histories without introducing lag due to an anti-alias filter is addressed by using multi-rate processing. The proposed CNN processes two windows of samples: one long window, which encompasses a long duration of deep past samples at a low sampling rate of 10 Hz, and one short window of the most recent input features at a higher rate of 50 Hz. This first window uses downsampling to compactly store a long time history of 2.00 s. The short window provides the estimator with a high-bandwidth version of the inputs for the most relevant samples in the time history, avoiding the lag and high frequency attenuation of the anti-aliasing filter.

Increasing the duration of the time history captures the repetitive patterns in the IMU data that emerge from walking more effectively, allowing the estimator to adapt to the unique gait patterns of the individual and changes in the walking speed.

However, increasing time history duration comes with the cost of computational complexity, impeding the real-time applicability of this algorithm. The solution is to downsample the inputs to reduce the number of samples required to represent the same time duration. Downsampling requires an anti-aliasing low-pass filter at the Nyquist frequency of the decimated sampling rate. This filter introduces phase delay and limits the rate of change of the input signals. The greater the downsampling factor, the lower the cutoff frequency of the anti-aliasing filter, and the more pronounced the lag effect is on the gait phase estimate. The delay is detrimental to the gait phase estimate since it introduces lag. In summary, downsampling is desirable to reduce the computational cost of estimating gait phase, but it negatively adds lag to the resulting estimate.

**Figure 4:**
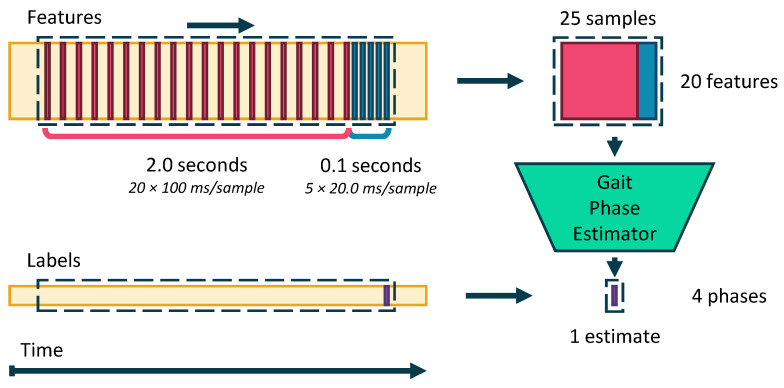
The input to the multi-rate estimator is a sliding window of the features sampled at a higher sampling rate for more recent data.

The input windows of data sampled at different rates are processed separately, and then combined in the deep layers of the network to form abstract feature maps. According to Figure 4, at each prediction time step (which occurs at a rate of 100 Hz), a window of 5 samples with inter-sample period of 20.0 ms (the short window) is taken from the most recent data, followed by a window of 20 samples with inter-sample period of 100 ms (the long window). The combined period of time captured in the window is 2.10 s over 25 samples. The target gait phase label for each window is the gait phase at the sample corresponding to the most recent sample in the input window.

The separation of data is necessary as the convolution kernels applied to each sampling rate do not necessarily generalize to data from the other sampling rates [43]. To avoid aliasing when downsampling to 50 Hz and 10 Hz for the short and long windows, respectively, the data streamed from the IMU are passed through a filter bank of second-order type-1 Chebyshev low-pass filters with cut-off frequencies of 25 and 5 Hz. The short window takes data from the first filter bank, and the long window takes data from the second.

**Figure 5:**
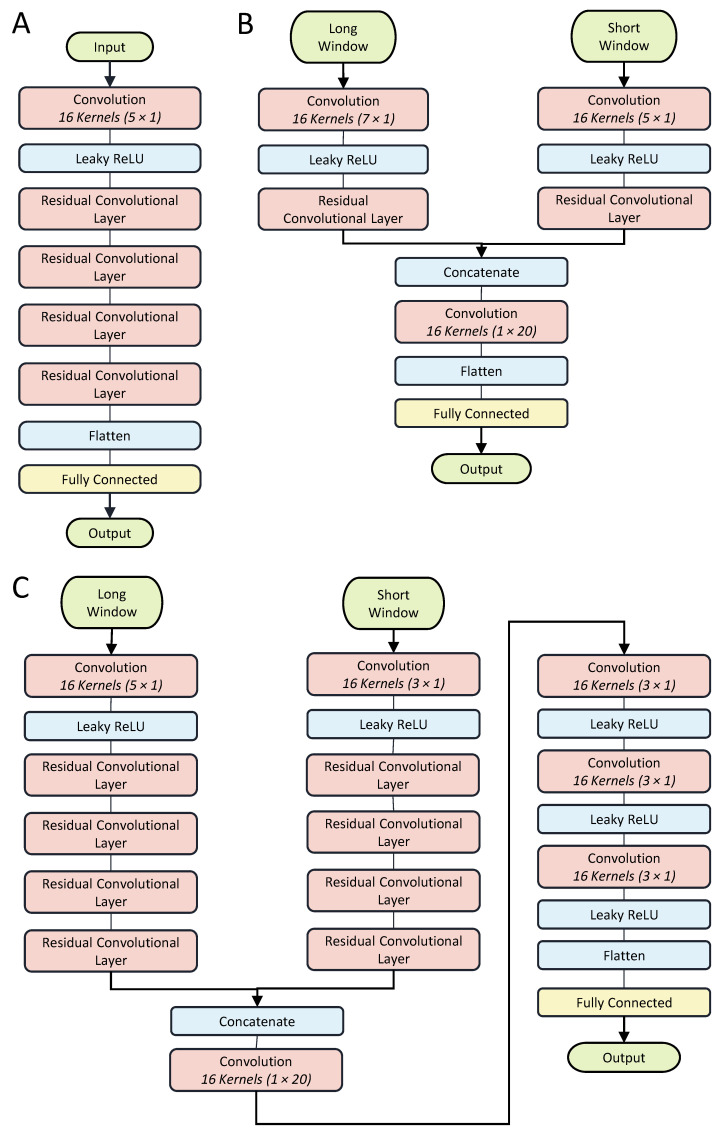
(**A**) The Single Head-4 model consists of 4 residual convolution blocks. (**B**) The Dual Head-1 model consists of 1 convolutional layer and 1 residual layer per head. The combined feature map is fed directly into a flattening and fully connected layer. (**C**) The Dual Head-4 model consists of 1 convolutional layer and 4 residual layers per head. The combined feature map is fed into 4 convolutional layers before the flattening and a fully connected layers.

Three different model architectures were compared to determine whether the multi-rate architecture and the deep layer architecture are justifiable. The first model is a single-rate estimator that only uses data sampled from the long window, shown in Figure 5A. The second model (Figure 5B) is a shallow dual-rate estimator that only uses 1 layer of residual blocks for each of the input windows. Figure 5C shows the last model, which is also a dual-rate model, but uses 4 residual layers per input window, as well as 2 convolutional layers after the concatenation. These models are referred to as the Single Head-4, Dual Head-1, and Dual Head-4 according to their input format and number of residual layers.

### 2.5. Network Training and Validation Procedure

One-subject-out cross-validation was used to determine the robustness of the estimator to new individuals. Gait phase and IMU data were partitioned into training, validation, and test, corresponding to the data for 14, 1, and 1 participants, respectively. The participant selected for the test set was permuted for each participant to obtain 16 datasets. Furthermore, the training was repeated with one-condition-out cross-validation. For each condition left out, we conducted 16 independent one-subject-out trials, and the statistics over those 16 tests were reported as the results for each condition out.

The model was trained with an ADAM optimizer [44] with an initial learning rate of 0.0012 and a batch size of 128. The learning rate was scheduled to drop by a factor of 0.2 every epoch. The training data were shuffled after every epoch.

The validation loss was computed every 800 iterations, which was approximately 7–8 times per epoch. The training was stopped when the validation loss stopped decreasing for 2 consecutive iterations, and the network with the best validation loss was used as the estimator for testing.

### 2.6. Performance Metrics

We measured the accuracy of the estimator using the root mean squared error between the estimated, $\hat{\phi }$, and target, ϕ, phases. Directly subtracting the estimated and target phases may lead to wraparound errors where two values on opposite sides of the transition boundary of the *atan2* function have a large difference, despite representing similar phases. To avoid this, the phase error, Δϕ, was computed for each leg using the formula for the signed angle between two 2-dimensional vectors.

(2) $$\Delta \phi_{k}=\Delta \phi_{k}-\Delta {\hat{\phi }}_{k}=\frac{1}{2\pi }atan2\left({\hat{y}}_{2}y_{1}-{\hat{y}}_{1}y_{2},{\hat{y}}_{1}y_{1}+{\hat{y}}_{2}y_{2}\right)$$

where $y_{1}$ and $y_{2}$ are the extended sinusoidal representation of ϕ, following the convention in Equation (1), and ${\hat{y}}_{1}$ and ${\hat{y}}_{2}$ are the sinusoidal components of $\hat{\phi }$. The spatial root mean squared error (sRMSE) of the predictions was computed for all *N* samples in the test dataset as follows:

(3) $$\mathrm{sRMSE}=\sqrt{\frac{1}{N}\sum_{k=1}^{N}{\left(\Delta \phi_{k}\right)}^{2}}.$$

The temporal error is the metric that is most commonly used in other studies to evaluate the latency of gait phase estimators when detecting key events such as heel strike [24,26,27]. The temporal distance was defined as the time between the estimated and actual heel strikes. The estimated heel strike time, $\hat{T}$, was matched to the closest actual heel strike at time *T*, and the difference was computed to obtain the temporal error. The temporal error was normalized by the actual duration of the previous stride. The temporal mean absolute error (tMAE) was calculated over all the $N_{s}$ estimated steps as follows:

(4) $$\mathrm{tMAE}=\frac{1}{N_{s}}\sum_{k=1}^{N_{s}}\left|\frac{T_{k}-{\hat{T}}_{k}}{T_{k}-T_{k-1}}\right|.$$

In order to compare the proposed estimators with the IMU-based estimator by Weigand et al. [31], the spatial mean absolute error (sMAE) of gait phase in linear form was computed for all walking conditions together, steady-state walking, and dynamic walking.

To compare the performance of the models in one-subject-out cross-validation scenario, a Friedman test with a level of significance of 0.05 was applied to observe whether there was a significant difference in the ranking of the model performances when trained on the same dataset. A two-tailed Wilcoxon signed rank test was then applied with a significance level of 0.05, with Bonferroni correction between each pair of models to determine if there was a significant change in error. In the one-section-out cross-validation scenario, a Kolmogorov–Smirnov (KS) test was applied with a significant level of 0.05 and Bonferroni correction to determine if there was a difference between the distributions of the errors for each model when a walking condition was removed from the training dataset.

The real-time capabilities of the three models were evaluated by running a MATLAB 2022b implementation of each model on 100 samples using an 8-core AMD Ryzen 7 4800H CPU (2.8 GHz) (Santa Clara, CA, USA) with 7-core Radeon GPU (1.6 GHz). The average and standard deviation of the computation time (CPU time) per sample over 10 repetitions was then computed for each model.

## 3. Results

### Comparison of Performance

In the subject-out cross-validation, the Dual Head-4 model performed the best in terms of temporal and spatial error. As shown in Table 1, the Dual Head-4 model had a lower average spatial and temporal error compared to the Single Head-4 model by 2.21% and 1.03%, respectively. The Single Head-4 and Dual Head-4 model had a smaller standard deviation of spatial and temporal error compared to the Dual Head-1 model.

The Friedman test showed a significant difference in the ranking of the three models when training on the same dataset, with *p*-values for spatial error and temporal error of $1.83\times 10^{-2}$ and $5.25\times 10^{-3}$, respectively. The Wilcoxon signed rank test showed a significant decrease of 1.96% and 2.22% in spatial error when comparing the Dual Head-1 and Dual Head-4 models to the Single Head-4 model with *p*-values of $5.57\times 10^{-4}$ and $3.24\times 10^{-4}$, respectively. The two dual head models did not have any significant difference in spatial error between them.

For temporal error, the Wilcoxon signed rank test showed that the Dual Head-4 model had a 1.03% smaller error than the Single Head-4 model when trained on the same data (*p* = $8.35\times 10^{-3}$). Unlike the test for the spatial error, the Dual Head-1 model did not have a significantly smaller temporal error compared to the Single Head-4 model.

**Figure 6:**
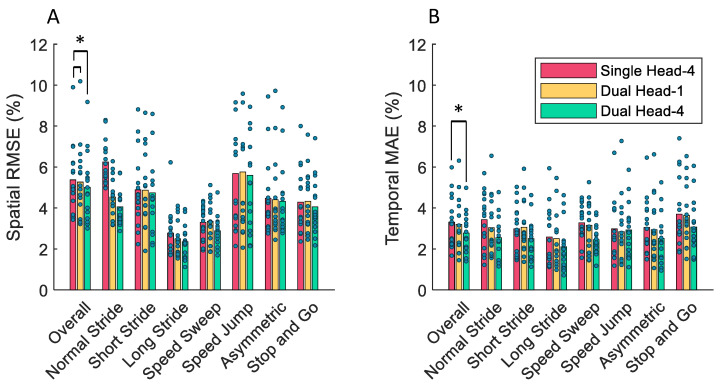
(**A**) The spatial and (**B**) temporal performance of the models in one-subject-out cross-validation separated by walking condition. Significant differences in the average performance rankings with a significance level of 0.05 between pairs of models trained on the same dataset are denoted by asterisks.

Figure 6A compares the spatial error of each model for each of the seven walking conditions separately. The Dual Head-4 produced the smallest average spatial error for every walking condition. Figure 6A shows that the three models had the lowest average spatial error when estimating the gait phase for the “Long Stride” and “Speed Sweep” walking conditions. In Figure 6B, the Dual Head-4 model also had the smallest average temporal error when considering each walking condition separately. Figure 6B shows relatively consistent temporal performance of all three models across all of the walking conditions.

Table 2 shows the performance of each model divided over steady-state walking conditions (conditions 1–4 in the experiment protocol) and the dynamic walking conditions (conditions 5–8) for comparison with other studies.

In the condition-out cross-validation experiment, the Dual Head-1 model had the smallest average spatial and temporal error. The KS test showed that the Dual Head-1 model had a significantly smaller spatial error than both the Single Head-4 and Dual Head-4 models when the normal walking condition was left out (both *p* = $9.38\times 10^{-4}$). Under all other walking conditions, there was no statistically significant difference in the spatial or temporal performance of the models. The mean and standard deviations of the performance metrics for each model are summarized in Table 3. The standard deviation was computed using all combinations of subjects and conditions left out.

**Figure 7:**
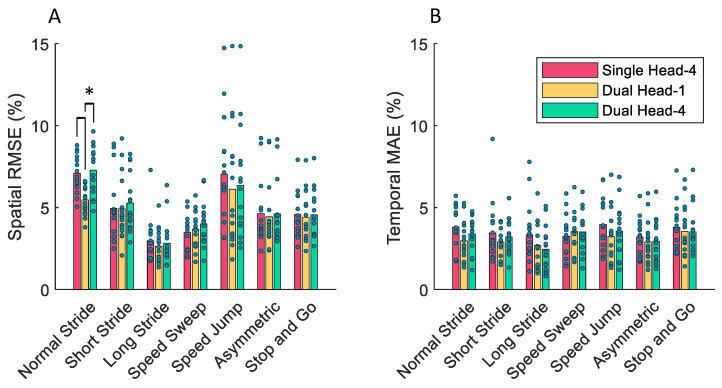
(**A**) The spatial and (**B**) temporal performance of the models in one-section-out cross-validation when each walking condition is left out. Comparisons with significantly different average errors using a level of significance of 0.05 are denoted by an asterisk.

The spatial and temporal errors of each model when the corresponding condition was left out from the training dataset are shown in Figure 7. Leaving out the “Normal Walking” or “Speed Jump” conditions from the training dataset led to the largest spatial errors for all three models.

For spatial error, the KS test showed that there was a significant difference in performance when leaving out the normal stride walking conditions for the Single Head-1 and Dual Head-4 models. On the contrary, there was no significant increase in temporal error compared to training on all of the data when leaving out each of the seven walking conditions separately.

In the real-time CPU test, the Single Head-4, Dual Head-1, and Dual Head-4 models took an average CPU time of 1.89 ± 0.0706 ms/sample, 2.27 ± 0.0551 ms/sample, and 2.75 ± 0.284 ms/sample, respectively. The model run times were below the 100 ms and 20 ms sampling periods of the single head and dual head models, respectively, suggesting the applicability of the algorithms in real-time applications such as control of lower-limb exoskeletons.

## 4. Discussion

### 4.1. Analysis of Accuracy and Generalizability

The motivation for a multi-resolution estimator comes from the observation that periodic patterns emerge more prominently in low-resolution analysis, and higher resolution data tends to have more variations [43]. In this application, the cyclic nature of walking results in quasi-periodic patterns in the IMU measurements at low resolutions; however, short duration, time-critical events, such as the heel strike, or sudden changes in gait patterns are more apparent at high resolutions.

A multi-rate estimator was chosen to balance the visibility of long-term gait patterns with the response time to changes in gait. The low sampling rate window efficiently captures long-term trends in the gait pattern to output a more accurate gait phase estimate. However, downsampling the input data requires low-pass filtering to prevent aliasing, which introduces phase delay and limits the rate of change of the input signals. The high sampling rate window provides the estimator with an increased data bandwidth, reducing the lag in the inputs.

The results of the Wilcoxon signed rank test show that both of the dual head models performed better in terms of spatial accuracy when training on the same data. Therefore, the 2.22% spatial and 1.03% temporal decrease in error from the single head to dual head model with the same network depth shows that the use of the multi-resolution signals is justified.

Only the Dual Head-4 had a significant improvement in temporal accuracy compared to the Single Head-4 model in the Wilcoxon signed rank test. Due to the fact that the Dual Head-1 and Dual Head-4 models take the same input data, it is reasonable to conclude that the increased complexity of the Dual Head-4 model is responsible for the increase in temporal performance. This improvement can be attributed to the deeper layers of abstraction in the Dual Head-4 model that are able to specialize on a wider range of gait patterns.

The difference between the tMAE of the Single Head-4 and Dual Head-4 models was 1.03%. For a stride duration of 1.44 s, representing the average stride duration across our participants at their preferred walking speed, a temporal error of 1.03% corresponded to a difference of 14.8 ms at the heel strike. To place this time period in context, we inspected the joint angles in a time period corresponding to 1.03% of the stride duration before and after the heel strike for all of the strides in the dataset. The maximum difference between the swing leg flexion angles during this period compared to the angles at the heel strike were, in the worst case, 6.08 degrees at the hip and 8.45 degrees at the knee. Such a significant joint angle error at the heel strike would be interpreted as a considerable tracking error by an impedance controller with moderate stiffness causing the application of significant corrective forces to the user’s legs.

The Dual Head-4 model had the smallest variance of temporal error across the 16 subjects; the temporal performance improvements generalized across individuals. Figure 6B shows that the temporal performance was also consistent across different walking conditions. The “Speed Jump” and “Stop and Go” conditions generally had the worst performance, as expected, because the changes in step frequency make the gait less predictable.

Overall, based on the subject-out cross-validation analysis, the Dual Head-4 performed best on average. The mean and variance of the spatial and temporal error for all of the models were larger for dynamic walking compared to steady state. This can be attributed to the increased variability of stride duration and asymmetry at higher walking speeds [45]. The periodicity of the gait is less predictable when the walking pace is changing, so the periodic events that constitute a well-defined gait phase are not as discernible. As expected, the Dual Head-4 model had the smallest mean error and variance in the spatial and temporal domains when considering the steady-state and dynamic walking conditions separately, showing that the performance improvements of the model are not limited to either consistent, stable gait patterns or variate walking conditions.

Table 3 shows that the average performance of the model does not decrease considerably when conditions are left out. This is further verified by the statistical analysis, which indicates that leaving out any of the walking conditions does not significantly degrade the temporal performance of the estimator. In most cases, leaving out walking conditions did not lead to a worse spatial error, except for leaving out the normal walking condition. This demonstrates the capability of the proposed models in accurate estimation of gait phase during abnormal or variable walking patterns, even if they have not been seen in the training process.

### 4.2. Comparison with State of the Art

The performance of the Dual Head-4 model is comparable with the recent methods that use joint angle encoders and foot contact sensors with a spatial RMSE of 5.00 ± 1.65% and temporal error of 2.78 ± 0.97%. These results show the feasibility of an IMU-based gait phase estimator, which is accurate across different gait conditions and neither imposes restrictions on the range of motion like joint encoders, nor requires foot contact sensors.

The CNN used by Kang et al., that takes hip joint angles measured by an encoder and an IMU situated on the trunk as inputs, had a spatial RMSE of 4.37 ± 0.68%, which was better than the proposed Dual Head-4 model by 0.63% on average [28]. When considering the steady-state and dynamic walking conditions separately, however, the Dual Head-4 model outperformed the CNN. During steady-state walking sections, the Dual Head-4 had an average RMSE of 3.95 ± 1.44%, which is 0.88% less than the average RMSE 4.83 ± 0.62% reported by Kang et al. [28]. In dynamic walking conditions, the Dual Head-4 model, which had an RMSE of 5.07 ± 2.00%, had a 3.04% lower average RMSE compared to the CNN model with an 8.11 ± 2.19% [28].

Hong et al. designed an estimator that uses the phase shift between the hip flexion angle and its integral to estimate the gait phase with a temporal error of 3.9% [24]. The temporal error of the Dual Head-4 is 1.1% less on average. The time delay neural network estimator presented by Shushtari et al. used joint angles, estimated from marker-based motion capture, as inputs, and had a spatial error of 1.74 ± 0.23% and a temporal error of 1.70 ± 0.54% [26], which, on average, is 3.26%, and 1.08% better in spatial and temporal error compared to the Dual Head-4 model. Lee et al. used the angular positions and velocities of the thigh and torso along with heel force sensors to predict heel strike timing, and achieved a spatial error of 1.67 ± 1.36%, which is 3.29% better on average compared to the Dual Head-4 model [27].

The methods compared above all require multiple types of sensors. In contrast, the proposed method uses only wearable IMUs, which are low-cost, accessible, lightweight, and well developed. Furthermore, unlike a joint encoder, IMUs do not require any fixtures that lie across the joint and may restrict the range of motion or mobility of the individuals. It is expected that the performance of the estimators with various sensors is better than that of the estimators that only use IMU measurements. The accelerations measured by the IMUs depend on the proximodistal position of the sensor on the thigh or shank. Also, IMUs do not directly measure spatial orientations, but, rather, estimate them using strapdown integration and sensor fusion algorithms.

Two previously proposed gait phase estimators that use only IMUs are a dense neural network, proposed by Weigand et al. [31], and an adaptive oscillator, by Zhang et al. [35]. Weigand et al. used an IMU mounted on the shank and a dense neural network to estimate gait phase when walking up to and ascending a staircase [31]. For the walking portion of the dataset, they obtained an sMAE of 2.0% in their lab environment, straight, level walking laboratory test, and an sMAE of 2.4% in their real-world environment. When they tested their estimator on a participant walking straight down a hallway, an increase in sMAE to 3.5% was reported. To compare the Dual Head-4 model, during steady-state walking, the Dual Head-4 model had an sMAE of 2.36 ± 0.824% and 2.85 ± 1.09% during dynamic walking. The results do not show any significant difference between the two estimators; however, the Dual Head-4 model was trained and evaluated on a more diverse range of walking conditions.

The method proposed by Zhang et al. uses a single IMU mounted to the thigh [35]. The algorithm is an adaptive optimizer that learns and predicts kinematic templates for the hip flexion angle, then uses dynamic time warping to match the current measured kinematics to the template to obtain a gait phase estimate [35]. Their overall spatial RMSE was 4.14±1.68%. The RMSE for steady-state level walking was 4.59±1.76%, and 6.77±2.29% when the speed was modulated. On average, the Dual Head-4 model performs 0.64% better in terms of spatial error during steady-state walking, and 1.70% under dynamic walking conditions. The performance increase of the Dual Head-4 model can be attributed to the multi-resolution inputs which handle both slow and fast dynamical changes in gait phase, as well as the greater range of participants and dynamic walking conditions in the dataset.

Given the variance of the performance results of the Dual Head-4 model, it is not justifiable to make claims on a performance improvement compared to other estimators that use only IMU sensors; however, this experiment used a much larger dataset of 16 participants under seven walking conditions that were designed to test a variety of gait conditions. Specifically, we trained and evaluated the estimator on asymmetric walking, changes in walking pace, and the transition from standing to walking, which were not considered in other studies on IMU-based estimators. The Dual Head-4 model had a better average performance on dynamic walking conditions compared to the CNN by Kang et al. [28] and the adaptive kinematic template method by Zhang et al. [35].

The Dual Head-4 model had a better temporal performance compared to the phase space estimator by Hong et al. [24]. Estimators that rely on hip angles alone for gait phase regression are limited by the assumption that the hip angle at the heel strike is consistent from stride to stride. The estimators in this experiment overcome this assumption by using the correspondence between peaks in the thigh and shank acceleration and the heel strike to accurately determine the gait phase near the heel strike.

### 4.3. Limitations and Potential Improvements

In this study, a range of controlled walking conditions were tested on a treadmill. For real-world applications, however, these walking conditions may not cover the full range of walking activities. In future work, the experiment should be replicated using overground walking conditions to verify the results of this study in more lifelike situations.

One of the unanswered questions from this study is whether wavelet or time-frequency analysis of the IMU data generates more informative features for gait phase estimation. In this study, the IMU data were low-pass filtered using filters that were designed to limit the bandwidth of the IMU signals. It is possible that separating the IMU data into frequency bands using a filterbank that is specifically tuned to extract features from the IMU could generate more informative, less noisy features. The risk with this method is that the filters require tuned parameters which may vary depending on the individuals and walking conditions, and poorly designed filters may result in worse performance than the proposed method.

Another modification that could possibly improve the performance of the estimator is to relabel the target gait phase using a piecewise linear function, as in [31,32]. These studies suggest that the target training labels for the swing and stance phases of gait should be labelled separately using linear functions with different rates. This modification to the training data increases the number of points in the gait cycle that have known, predetermined gait phase values: the heel-strike corresponding to a gait phase of 0%, and the toe-off corresponding to a gait phase of 63% [31]. This helps maintain more consistent gait phase values for abnormal strides in the dataset, leading to possibly more accurate predictions after training.

## 5. Conclusions

In this paper, a real-time gait phase estimator was proposed that uses only IMU sensor data. A deep multi-resolution CNN, the Dual Head-4 model, was trained on a dataset that included 16 participants walking under a range of speeds between 0.1 to 1.9 m/s, stride lengths, asymmetries, and intermittent speed changes. The Dual Head-4 model was compared with two other models, with a single resolution input and shallow layer architecture, respectively. A Wilcoxon signed rank test showed that incorporating data sampled at different rates into the model inputs significantly improves the spatial accuracy of the estimator, while a deep layer architecture improves the temporal accuracy. The average sRMSE was 5.00±1.65% and the tMAE was 2.78±0.96%. In steady-state walking conditions, the estimator had a sRMSE of 3.95±1.44% and a tMAE of 2.34±0.70%. In dynamic walking conditions, the estimator had a sRMSE of 5.07±2.00% and tMAE of 2.90±1.22%. A Kolmogorov–Smirnov test showed that there were no significant changes in spatial or temporal performance when testing on left-out abnormal walking conditions, suggesting that the estimator is robust to new individuals, gait patterns, and walking speeds.

## Figures and Tables

**Table 1:** One-subject-out cross-validation results.

Model	Spatial RMSE (%)	Spatial MAE (%)	Temporal MAE (%)
Single Head-4	7.22±1.22	3.18±0.93	3.81±1.23
Dual Head-1	5.26±1.80	3.14±0.99	3.20±1.29
Dual Head-4	5.00±1.65	2.94±0.81	2.78±0.97

**Table 2:** Steady-state and dynamic model performance results.

Model	Spatial RMSE (%)		Spatial MAE (%)		Temporal MAE (%)	
	Steady State	Dynamic	Steady State	Dynamic	Steady State	Dynamic
Single Head-4	4.29±1.47	5.48±2.10	2.78±1.02	3.08±1.18	2.88±1.02	3.54±1.04
Dual Head-1	4.14±1.38	5.28±2.28	2.59±0.99	3.09±1.26	2.87±1.06	3.21±1.55
Dual Head-4	3.95±1.44	5.07±2.00	2.36±0.82	2.85±1.09	2.34±0.70	2.90±1.22

**Table 3:** One-condition-out cross-validation average results.

Model	Spatial RMSE (%)	Temporal MAE (%)
Single Head-4	4.97±2.35	3.54±1.48
Dual Head-1	4.53±2.18	3.10±1.33
Dual Head-4	4.99±2.31	3.23±1.31

## Data Availability

The data presented in this study are available on request from the corresponding author.

## Abbreviations

The following abbreviations are used in this manuscript:

IMU	Inertial measurement unit
CNN	Convolutional neural network
iSCI	Incomplete spinal cord injury
AFO	Adaptive frequency oscillator
EKF	Extended Kalman filter
ANN	Artificial neural network
RNN	Recurrent neural network
LSTM	Long short-term memory network
TDNN	Time-delay neural network
MSE	Mean squared error
RMSE	Root mean squared error
sRMSE	Spatial root mean squared error
sMAE	Spatial mean absolute error
tMAE	Temporal mean absolute error
KS	Kolmogorov–Smirnov
